# Outcomes of total arch replacement and frozen elephant trunk in acute aortic syndrome

**DOI:** 10.3389/fcvm.2025.1686781

**Published:** 2026-01-02

**Authors:** Étienne Fasolt Richard Corvin Meinert, Jamila Kremer, Mina Farag, Anna Lassia Meyer, Bashar Dib, Matthias Karck, Rawa Arif

**Affiliations:** Department of Cardiac Surgery, Heidelberg University Hospital, Heidelberg, Germany

**Keywords:** frozen elephant trunk, total arch replacement, aortic dissection, acute aortic syndrome (AAS), GERAADA score

## Abstract

**Objectives:**

There are several studies from all over the world reporting on frozen elephant trunk implantation and total arch replacement in acute aortic syndrome demonstrating mostly favourable outcomes. Most of these studies present younger study populations carrying a rather low perioperative risk for adverse outcomes. Herein, we present our single centre experience with the frozen elephant trunk procedure in patients with acute aortic syndrome. The patients in this cohort carried a rather high perioperative risk. A considerable number of patients had undergone resuscitation, presented with neurological disorders or presented with malperfusion syndrome. We demonstrate that favourable outcomes are achievable in such high-risk patients using the frozen elephant trunk technique.

**Methods:**

All patients who underwent frozen elephant trunk implantation in a setting of acute aortic syndrome between March 2008 and March 2023 were included in this retrospective study.

**Results:**

Overall, 90 patients underwent frozen elephant trunk implantation due to acute aortic syndrome. Mean age was 60.0 (±11.6) years, 74 patients (82%) were male. All had extensive aortic pathologies with involvement of the aortic arch, supraaortic vessels or descending aorta. 27 patients (30%) presented with neurological disorders, including aphasia, hemiparesis, paraparesis and coma. Predicted 30-day mortality by the so called GERAADA score was 23.9% on average. In our cohort, we observed an actual 30-day mortality of 17.4%. Postoperatively, neurological disorders were observed in 34 patients (38%). Aortic redo surgery was required in 8 patients (9%). Several preoperative and intraoperative parameters were tested for prediction of 30-day-survival. Preoperative hemiparesis (*p* = 0.012), visceral malperfusion (*p* = 0.004) and preoperative resuscitation (*p* = 0.003) served as significant predictors in a multivariable cox regression.

**Conclusions:**

The recent adaptation of frozen elephant trunk implantation in acute aortic syndrome led to an improved outcome. Overprediction trend of early mortality by the GERAADA score and a low rate of aortic redo surgery in the long-term course support this idea.

## Introduction

By now there are several studies from all over the world reporting on frozen elephant trunk (FET) implantation and total arch replacement (TAR) in acute aortic syndrome demonstrating mostly favourable outcomes (1–5). Quite some of these studies however, present younger study populations carrying a rather low perioperative risk for adverse outcomes. Herein, we present our single centre experience with the FET procedure in patients with acute aortic syndrome. The patients in this cohort carried a rather high perioperative risk. The average predicted 30-day mortality risk by the GERAADA score (6, 7) was 23.9%. Specifically, 6% of patients had undergone resuscitation, 30% of patients presented with neurological disorders, 43% presented with any malperfusion syndrome, 16% had severe aortic regurgitation, catecholamines at referral was noted in 10% of patients and intubation and assisted ventilation at referral were noted in 12% of patients. We demonstrate that favourable outcomes are achievable in such high risk patients using the FET technique in acute aortic syndrome.

## Methods

This retrospective study complies with the declaration of Helsinki and was approved by institutional review board of the research ethics committee of Heidelberg University, Heidelberg, Germany (S-661/2023) on 26th of January 2024. Written patients informed consent was waived by the committee.

In this single centre retrospective study, we analysed outcomes of FET implantation and TAR for acute aortic syndrome involving the ascending aorta. All patients were included who had undergone such a procedure between 1st of January 2008 and 31st of March 2023.

Perioperative data were acquired through available routine documentation records such as surgery reports or patient records. Follow-up was until 1st of July 2023 i.e., patient data were considered up until this day. All data were anonymised.

Primary endpoint was 30-day mortality. The secondary endpoint was stroke resulting in neurological residues. Median time to censoring was 161 days and mean time to censoring was 637 days. Long term follow up was only partially available, so that overall long term survival reporting would be biased.

Indication for FET implantation in acute aortic syndrome was extension of the pathology into the aortic arch and descending aorta or presence of entry tears in the aortic arch or proximal descending aorta. Also, the indication was made on a case-by-case basis at the surgeon's discretion, considering the general condition and risk factors of the patient.

Surgical access was median sternotomy in all patients. Several cannulation strategies were applied owing to different extensions of aortic pathologies. These included arterial cannulation of the true lumen of the ascending aorta under transoesophageal echocardiographic guidance, or cannulation of the right or left axillary artery or femoral arteries. The arterial cannulation strategy was at the surgeon's discretion. Basically, the strategy was to safely cannulate the true lumen to provide adequate perfusion. Some surgeons may have been more comfortable with certain ways of cannulation and opted for them in an emergent scenario. The right atrial appendage or a femoral vein were used for venous cannulation. Opening of the aortic arch was performed under mild to moderate hypothermic circulatory arrest with selective antegrade cerebral perfusion. The landing zone (i.e., distal anastomosis in zone 2 or zone 3) was zone 3 for the vast majority of patients. As distal anastomosis in zone 2 has only be performed since 2021 by a few surgeons at our institution, these data were not routinely collected, since they were not coherently reported. Bretschneider cardioplegia was used for myocardial protection.

Two types of frozen elephant trunk prostheses were used. Between 2008 and 2015 an Evita Jotec prosthesis with a stent length of 13 cm was used in 26 patients (29%). From 2016 onward, a Thoraflex Hybrid prosthesis with a stent length of 10 cm was used in 64 patients (71%).

Statistical tests were performed as indicated. *P* values are two-tailed. Basically, *p* values lower than 0.05 were considered statistically significant. Statistical analyses were performed using R version 4.3.0 or newer. For descriptive statistics, the “freqtables” package was used. The cumulative hazard plot using the Kaplan Meier method was created using the “survminer” and “ggplot2” packages. The parallel coordinate plot depicting the neurological outcomes was created using the “ggparallel” package. The univariate and multivariable regression analyses were performed using the “ggsurvfit”, “Survival” and “survminer” packages. Univariable cox regression was performed to screen for suitable variables for a multivariable model. We then selected four preoperative variables based on *p* value <0.1, hazard ratio >2 and presumed clinical relevance, aiming to avoid clinically strongly interrelated variables. Furthermore, we tested the selected variables for the multivariable model for multicollinearity using the “car” package. Variance inflation factors between 1 and 5 were assumed to represent no to moderate multicollinearity. Results were depicted in forest plots using the “ggsurvfit” package.

## Results

Between 1st of January 2008 and 31st of March 2023, 90 patients underwent FET implantation in the setting of acute aortic syndrome at our institution (Table 1). Pathologies included 81 cases (90%) of aortic dissections type A (AADA) and less frequently acute aortic dissection type B, non-A-non-B aortic dissection, intramural haematoma and penetrating aortic ulcer.

**Table 1:** Descriptive statistics of preoperative variables.

Variable		Total	Percentage	Mean	Median	Standard deviation
All		90	100%			
Sex	Male	74	82%			
	Female	16	18%			
Age [years]		60.0		60.0	59.9	11.6
Aortic valve regurgitation	None	29	32%			
	I-II	39	43%			
	III-IV	14	16%			
	NA	7	8%			
Neurologic state	Coma	1	1%			
	Hemiparesis	10	11%			
	Paraparesis	6	7%			
	Other	10	11%			
	None	54	60%			
	NA	9	10%			
Malperfusion	Coronary	4	4%			
	Cerebral + Peripheral	3	3%			
	Coronary + Peripheral	1	1%			
	Renal + Peripheral	2	2%			
	Cerebral	6	7%			
	Spinal	2	2%			
	Visceral	4	4%			
	Renal	8	9%			
	Peripheral	11	12%			
	None	37	41%			
	NA	12	13%			
Extension of dissection	Aortic Arch	1	1%			
	Descending Aorta	3	3%			
	Aortic Arch + Descending Aorta	23	26%			
	Aortic Arch + Supraaortic Vessels + Descending Aorta	58	64%			
	Aortic Arch + Supraaortic Vessels	1	1%			
	Supraaortic Vessels + Descending Aorta	3	3%			
	NA	1	1%			
Entry tear arch	N	42	47%			
	Y	38	42%			
	NA (0)	10	11%			
Catecholamines at referral	N	70	78%			
	Y	9	10%			
	NA (0)	11	12%			
Resuscitation before surgery	N	84	93%			
	Y	5	6%			
	NA (0)	1	1%			
Pericardial effusion or tamponade	N	43	48%			
	Y	38	42%			
	NA (0)	9	10%			
Intubation/ventilation at referral	N	70	78%			
	Y	11	12%			
	NA (0)	9	10%			
GERAADA score [%]		23.9		23.9	20.1	13.3
BMI [kg/m^2^]		27.9		27.9	27.5	4.5
Lactate [mg/dL]		23.8		23.8	14.6	23.2
Previous cardiac surgery	N	85	94%			
	Y	4	4%			
	NA	1	1%			
Diabetes mellitus	N	85	94%			
	Y	4	4%			
	NA	1	1%			
Hypertension	N	28	31%			
	Y	58	64%			
	NA	4	4%			
Smoking	Never	58	64%			
	Ever	24	27%			
	NA	8	9%			
Diagnosis	AADA	81	90%			
	AADB	2	2%			
	NANB	2	2%			
	IMH	4	4%			
	PAU	1	1%			
	NA	0	0%			

Mean age was 60.0 years (SD: 11.6), 74 patients (82%) were male. Several preexisting conditions were recorded: 4 patients (4%) had diabetes, 58 patients (64%) had hypertension, 4 patients (4%) had previously undergone cardiac surgery, 24 patients (27%) had a history of smoking, mean body mass index was 27.9 kg/m^2^ (SD: 4.5).

All had extensive aortic pathologies with involvement of the aortic arch, supraaortic vessels or descending aorta. The following extensions of aortic pathology were most common. Involvement of the aortic arch and supraaortic vessels and descending aorta was noted in 58 cases (64%). Involvement of the aortic arch and descending aorta was noted in 23 cases (26%).

Preoperative state varied considerably. 27 patients (30%) presented with neurological disorders, including hemiparesis (10 cases or 11%), paraparesis (6 cases or 7%), coma (1 case or 1%) and others such as aphasia (10 cases or 11%). Notably, 5 patients (6%) had undergone preoperative resuscitation. Clinical or radiological evidence of cerebral, spinal, coronary, renal visceral or peripheral malperfusion was evident in 41 patients (46%). Aortic regurgitation was present in 53 patients (59%), 14 of whom (16%) had severe aortic regurgitation. Catecholamines at referral was noted in 9 patients (10%). Intubation and assisted ventilation at referral were noted in 11 patients (12%). Pericardial effusion or pericardial tamponade at referral were noted in 38 patients (42%). Mean lactate level at referral was 23.8 mg/dL (SD 23.2).

Predicted 30-day mortality by the GERAADA score was 23.9% (SD: 13.3) on average.

Several technical intraoperative parameters were recorded (Table 2). Mean total procedure time was 445 min (SD: 117). Mean cardiopulmonary bypass time was 289 min (SD: 83). Mean aortic cross clamp time was 176 min (SD: 47). Mean circulatory arrest time was 75 min (SD: 36). Mean lowest body temperature was 23.8 °C (SD: 2.5).

**Table 2:** Descriptive statistics of intraoperative variables.

Variable		Total	Percentage	Mean	Median	Standard deviation
All		90	100%			
Arterial cannulation site	Aorta	33	37%			
	Right Axillary Artery	25	28%			
	Left Axiallary Artery	1	1%			
	Right Femoral Artery	27	30%			
	Left Femoral Artery	2	2%			
	NA	2	2%			
Total procedure time [min]		445		450	416	117
Cardiopulmonary bypass time [min]		289		289	273	83
Aortic crossclamp time [min]		176		176	176	47
Circulatory arrest time [min]		75		75	78	36
Lowest body temperature [°C]		23.8		23.8	24.0	2.5
Frozen elephant trunk type	Jotec	26	29%			
	Thoraflex	64	71%			
	NA	0	0%			
Concomitant aortic valve procedure	None	58	64%			
	Aortic Valve Replacement	4	4%			
	Bentall	10	11%			
	David	11	12%			
	Other	7	8%			
	NA	0	0%			
Concomitant CABG	N	82	91%			
	Y	8	9%			
	NA	0	0%			
Concomitant TEVAR	N	83	92%			
	Y	7	8%			
	NA	0	0%			
TEVAR within 30d	N	79	88%			
	Y	11	12%			
	NA	0	0%			
Diagnosis	AADA	81	90%			
	AADB	2	2%			
	NANB	2	2%			
	IMH	4	4%			
	PAU	1	1%			
	NA	0	0%			

Two types of frozen elephant trunk prostheses were used. Between 2008 and 2015 an Evita Jotec prosthesis with a stent length of 13 cm was used in 26 patients (29%). From 2016 onward, a Thoraflex Hybrid prosthesis with a stent length of 10 cm was used in 64 patients (71%).

Owing to the extent of aortic pathology, different arterial cannulation strategies were applied. In 33 cases (37%) direct cannulation of the ascending aorta was performed. Other sites included the right axillary artery in 25 patients (28%), the right femoral artery in 27 patients (30%), and less commonly the left femoral artery in 2 patients (2%) or the left axillary artery in 1 patient (1%).

Concomitant aortic valve procedures were performed in 32 patients (35%), including aortic valve replacement in 4 patients (4%), Bentall procedure in 10 patients (11%), David procedure in 11 patients (12%) and others such as partial aortic root replacement in 7 patients (8%).

Moreover, 8 patients (9%) underwent concomitant coronary artery bypass grafting and 7 patients (8%) underwent concomitant thoracic endovascular aortic repair.

**Figure 1:**
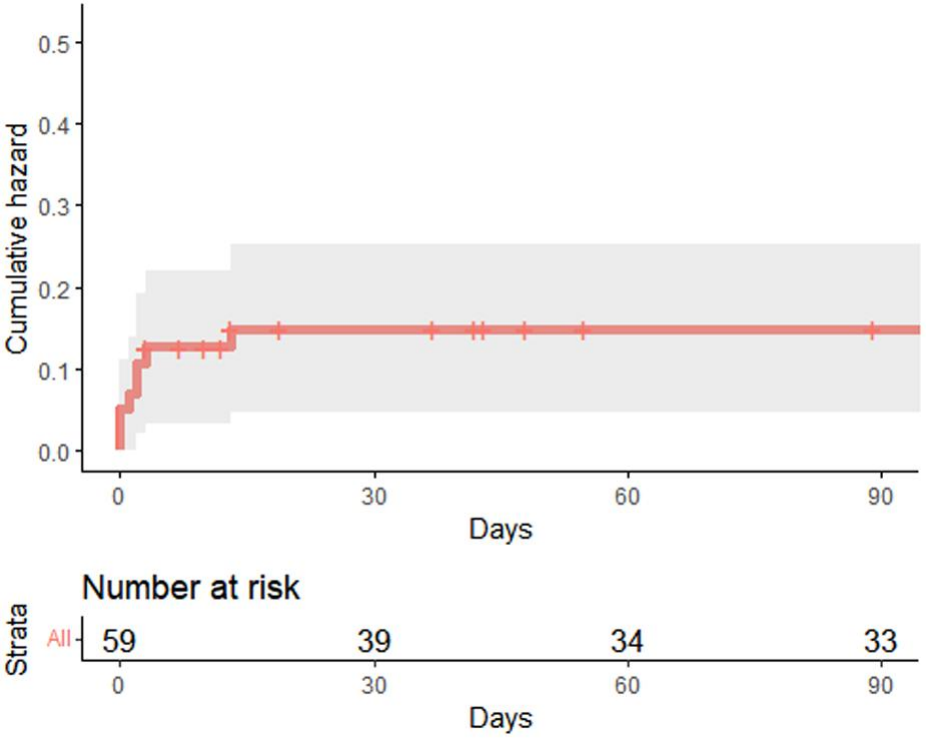
Cumulative hazard plot showing results of 90-day survival analysis.

In our cohort, we observed a 30-day mortality of 17.4% (SEM: 4.1) (Table 3) (Figure 1). Causes of 30-day mortality included: cardiogenic shock, rupture of the descending aorta, brain death or multi-organ-failure. There was a trend of overpredicting 30-day mortality using the GERAADA Score [mean: 23.9% (SD: 13.3)], but without a statistically significant difference (unpaired *t*-test *p* = 0.115).

**Table 3:** Descriptive statistics of postoperative variables.

Variable		Total	Percentage
All		90	100%
Neurologic state	Coma	7	8%
	Tetraparesis	4	4%
	Hemiparesis	8	9%
	Paraparesis	10	11%
	Other	5	6%
	None	48	53%
	NA	8	9%
Postoperative haemodialysis	N	68	76%
	Y	21	23%
	NA	1	1%
Reexploration	Redo thoracotomy	10	11%
	Inferior pericardiotomy	3	3%
	None	77	86%
	NA	0	0%
Aortic redo surgery	N	82	91%
	Y	8	9%
	NA	0	0%

**Figure 2:**
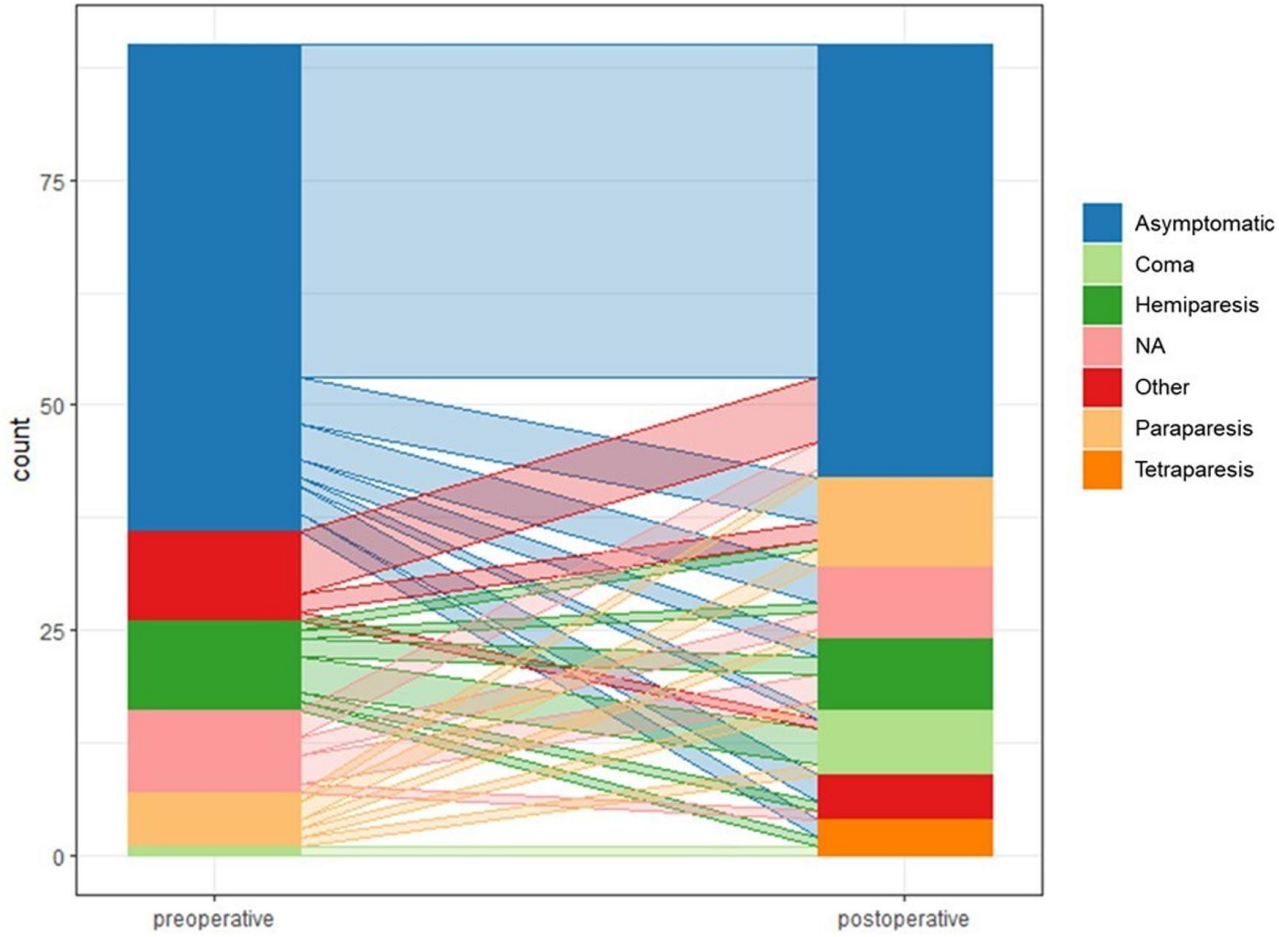
Parallel coordinate plot showing individual development of neurological status (preoperative and postoperative). On the left side, distribution of preoperative neurological status can be observed. On the right side, distribution of postoperative neurological status can be observed. The transition from preoperative to the postoperative neurological status can be appreciated through the transparent lines.

Following surgery, neurological disorders were observed in 34 patients (38%) (Figure 2). In detail, coma was observed in 7 patients (8%), tetraparesis was observed in 4 patients (4%), hemiparesis was observed in 8 patients (9%), paraparesis was observed in 10 patients (11%) and 5 patients (6%) experienced other neurological disorders such as aphasia.

Reexploration for bleeding was required in 13 patients (14%). Postoperative haemodialysis was required in 21 patients (23%).

Aortic redo surgery was required in 8 patients (9%). Long term follow up was only partially available, so that overall long term survival reporting would be biased.

Since 2017, favourable results and the introduction of a new type of prosthesis have led to an increased utilization of the approach. 26 patients (29%) received a FET before 2017 and 64 patients (71%) after that time point. This likely reflects an institutional learning curve with more surgeons adopting the technique. Also, the newer device appears to be more straightforward to use.

**Figure 3:**
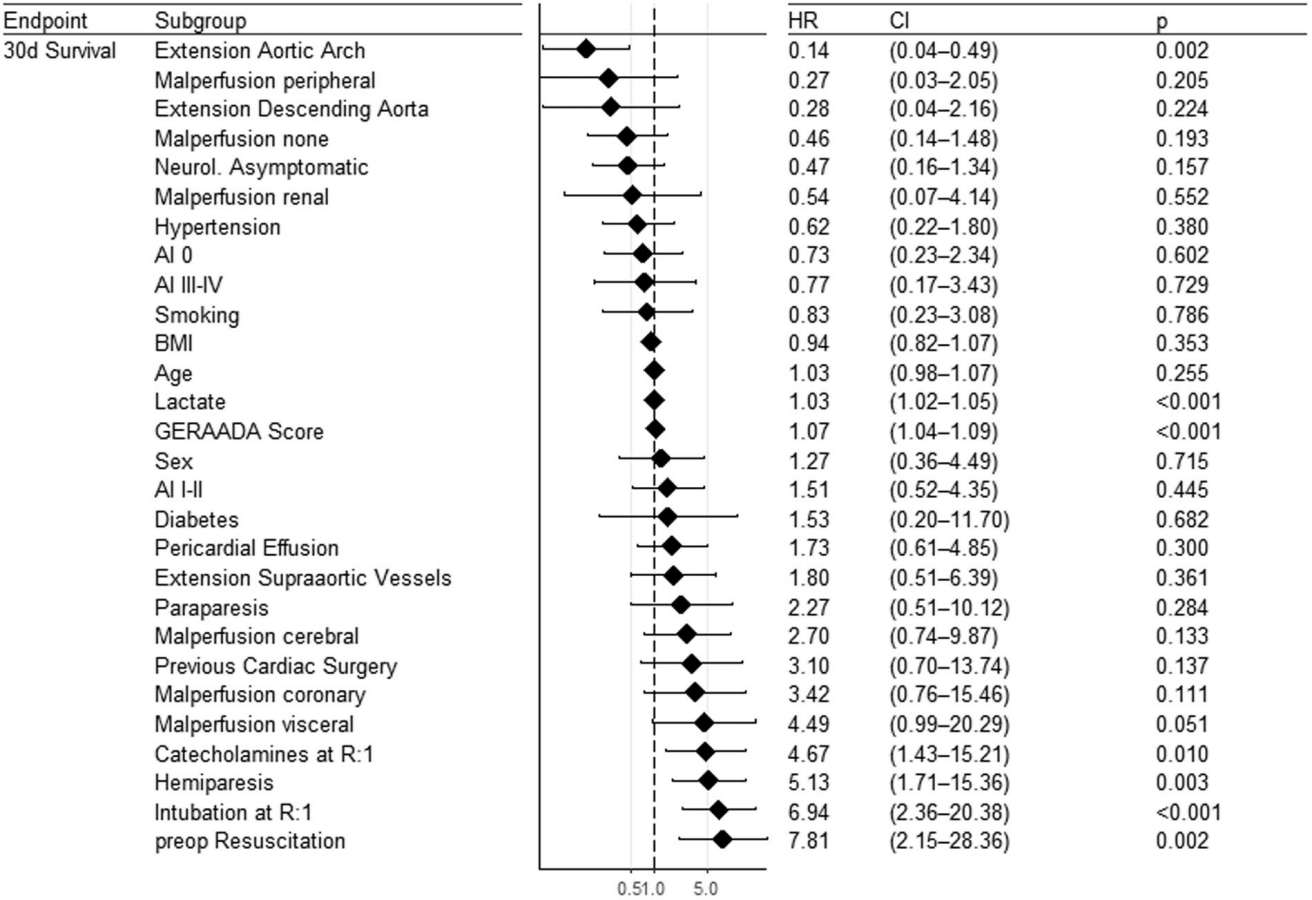
Forest plot showing results of univariate cox regression for preoperative parameters.

**Figure 4:**
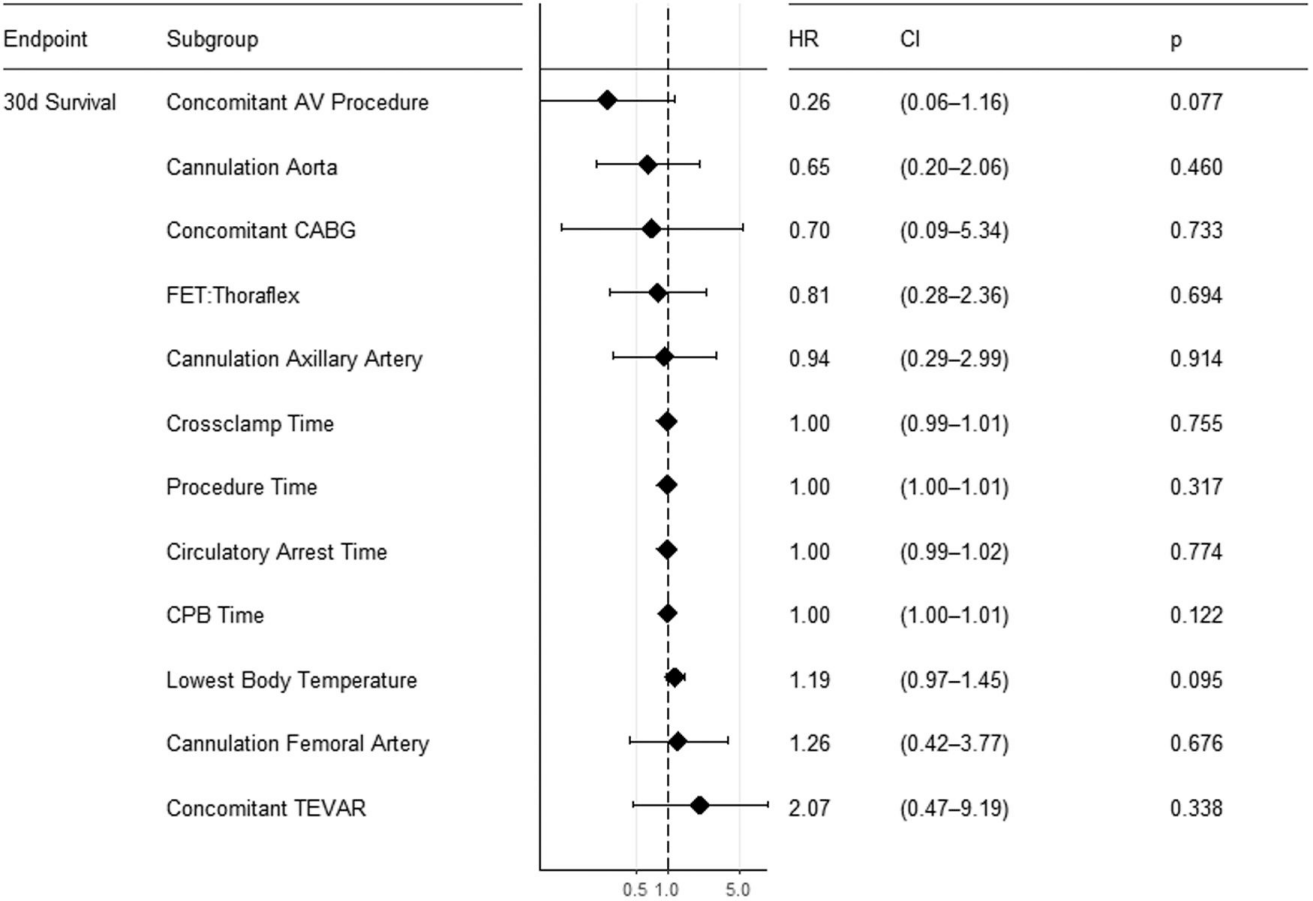
Forest plot showing results of univariate cox regression for intraoperative parameters.

**Figure 5:**
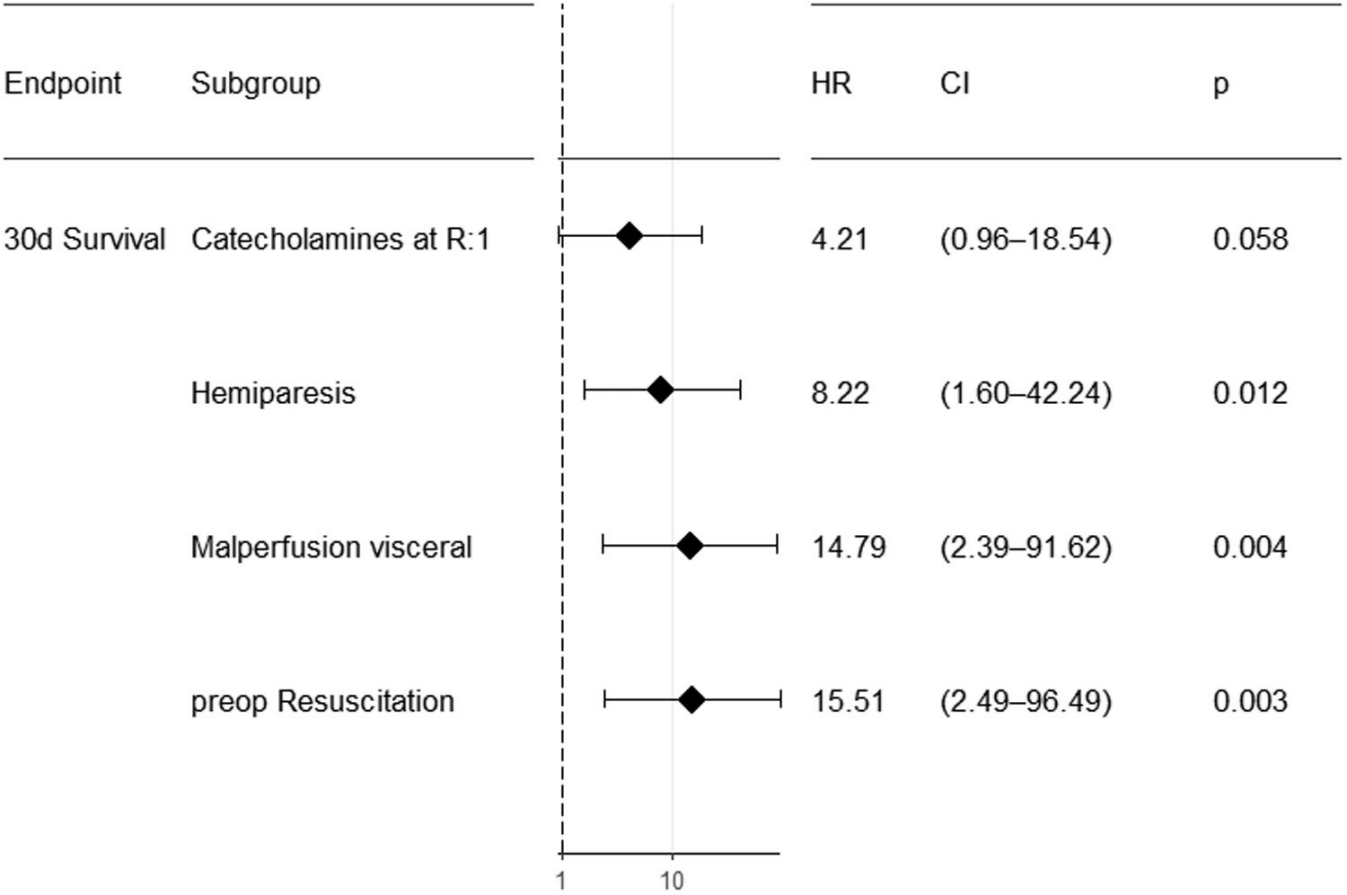
Forest plot showing results of multivariable cox regression for preoperative parameters.

Several preoperative and intraoperative parameters were tested for prediction of 30-day survival (Figures 3–5). Reasonable candidate variables, likely not being clinically interrelated, proving significant in the univariate model were selected for a multivariable regression analysis. In this multivariable cox regression model, preoperative hemiparesis (*p* = 0.012), visceral malperfusion (*p* = 0.004) and preoperative resuscitation (*p* = 0.003) served as significant predictors, while catecholamines at referral did not (*p* = 0.058). In view of only 10 events in 90 patients we additionally tested for multicollinearity using the variance inflation factor (vif). It was between 1.01 and 1.03 for all tested variables in the multivariable regression model, indicating a low level of multicollinearity (catecholamines at referral vif = 1.027; preoperative hemiparesis vif = 1.017; visceral malperfusion vif = 1.024; preoperative resuscitation vif = 1.024). Variables such as cardiopulmonary bypass time or concomitant procedures had no significant influence on survival.

## Discussion

### Main findings

Our study demonstrates that by employing the frozen elephant trunk procedure, favourable short-term outcomes may be achieved in patients presenting with acute aortic syndrome, bearing a high-risk profile. The GERAADA score gives a good overview of the preoperative mortality risk in such patients (6). Predicted 30-day mortality by the GERAADA score was 23.9% on average. Specifically, mean age was 60 years. All had extensive aortic pathologies with involvement of the aortic arch, supraaortic vessels or descending aorta. 30% of patients presented with neurological disorders. 6% of patients had undergone preoperative resuscitation. Clinical or radiological evidence of any malperfusion was observed in 46% of patients. 16% had severe aortic regurgitation. 10% required catecholamines at referral and 12% required. intubation and assisted ventilation at referral Pericardial effusion or pericardial tamponade at referral were noted in 38 patients (42%).

Still, relatively favourable short-term outcomes could be achieved. We observed an actual 30-day mortality of 17.4%. Postoperative neurological disorders were observed in 38% of patients. There might be a chance that neurological symptoms may have regressed following rehabilitation in several patients.

### Comparison

The GERAADA score predicted a 30-day mortality of 23.9% in our cohort. Although not statistically significant (*p* = 0.115), there is a visible trend of overprediction of mortality for our study population with an actual 30-day mortality of 17.4%. Several studies have made efforts to validate the GERAADA score with satisfying results (8, 9), so that its use has received a class IIa recommendation in the latest EACTS/STS Guidelines on acute aortic syndromes (7). This trend towards overprediction in our cohort is also an indicator that FET implantation may improve short term prognosis in such high-risk patients.

Several studies on FET implantation in AADA exist. Usually, studies from western countries report on older patients, with a higher risk profile and worse outcomes than studies from eastern Asia.

For instance, in a multinational multicentre (2) study including 471 patients who had received FET implantation for AADA between 2000 and mean age was 50 years, 5.9% had a cerebrovascular accident and short-term mortality was 11.3%, rate of permanent neurological deficit was 10.1% and rate of spinal-cord injury was 6.3%. The patients in this cohort are younger than in our cohort (mean age 60 years). Relevant variables such as preoperative hemiparesis or preoperative resuscitation or preoperative malperfusion syndromes (6) were not reported, but it may be fair to assume that the preoperative risk of the patients in this multicentre study was rather low.

In a German single-centre study (3) including 115 patients who received FET implantation for AADA between 2013 and 2019 mean age was 57 years, 20.9% had presented with a cerebral neurological deficit and 42.6% had presented with signs of preoperative malperfusion. A 30-day mortality rate of 10.4% and a rate of disabling stroke of 19.1% and a permanent paraplegia rate of 0.9% was reported. The risk profile of this cohort seems to be comparable to our study group, although our cohort was slightly older and presented with neurological disorders more frequently.

A Japanese single-centre study (4) including 139 patients who received FET implantation for AADA between 2007 and 2018 compared FET vs. no FET in AADA. Mean age was 59.6 years, preoperative rate of coma or hemiparesis was 9.4%. Moreover 50.4% presented with signs of malperfusion. Details on malperfusion syndrome or preoperative resuscitation were not reported. 30-day mortality was reported as 1.4% and postoperative neurological dysfunction as 5.0%. This study appears to present a study population with a risk profile comparable to our study. The huge difference in short-term outcomes is difficult to explain.

In a large Chinese single centre study (5) including 1,225 patients who received FET implantation for AADA between 2010 and 2017 mean age was 47.0 years. In this cohort the authors presented a 30-day mortality rate of 9.4%, a stroke rate of 2.7% and a paraplegia rate of 4.3%. The preoperative risk profile is not clearly reported for this study population, but it may be lower than in our cohort. Another aspect might be the huge case volume of this Chinese centre. It has been shown that high case volumes per surgeon (10, 11) or centre (12) positively affect outcome in thoracic aortic surgery.

### Predictors

In our cohort, several variables could be identified as predictors of unfavourable short-term outcome. We found preoperative hemiparesis (*p* = 0.012), visceral malperfusion (*p* = 0.004) and preoperative resuscitation (*p* = 0.003) to predict 30-day mortality in a multivariable cox regression. Our model is only based on 90 subjects. External validity could not formally be tested in lack of publicly available data sets on the subject. External validity however can be implied, since the variables predicting short term survival also appear to play a role in larger studies, such as the GERAADA study (6).

Variables such as cardiopulmonary bypass time, type of prosthesis or concomitant procedures had no significant effect on survival.

Interestingly, a comparison of the Evita Jotec and Thoraflex Hybrid prostheses in a multicentre retrospective study involving 88 patients, no survival difference could be found (13), which is in line with our results, revealing no effect on survival.

Moreover, concomitant procedures, especially concomitant root procedures did not lead to an increased mortality. Especially, no patient out of 11 patients (12%) who received concomitant David procedure died within 30 days. This might in part be explained by the idea, that the combination of FET implantation and David procedure would mainly be undertaken in rather healthy individuals with a lower risk profile, although our study cohort is too small to test this hypothesis. This finding is in line with another study, showing no increased risk of concomitant aortic root procedures in the same setting (3).

### Limitations

This study has several drawbacks, limiting its significance. It is retrospective in nature, has no comparison group and involves a rather low number of cases.

Also, follow-up data are incomplete. The rate of any aortic reoperation including open aortic repair and endovascular procedures was 9% in our cohort. Data on postoperative status would routinely be collected after six months. Patients with issues, such as persisting false lumen perfusion in their postoperative CT scan would commonly be seen in a vascular surgery outpatient consultation with regular follow-up scans occasionally prompting secondary operations. Long term follow up was only partially available, so that overall long term survival reporting would be biased. Any long-term data of this study need to be interpreted with caution due to incomplete follow-up.

The timeframe of the study of 15 years is quite large. On the other hand, perioperative data were thoroughly collected, and the study population carries a rather high- risk profile making our findings more applicable to a real-world setting.

### Conclusion

In summary, our study demonstrates satisfying results of FET implantation in acute aortic syndromes, even in a rather high-risk study group. The GERAADA score trended to overpredict short term mortality by a slight margin. Preoperative lactate levels, preoperative hemiparesis and preoperative resuscitation served as predictors of 30-day mortality in a multivariate cox regression. However, increased cardiopulmonary bypass time or concomitant procedures did not increase the operative risk.

## Data Availability

The datasets presented in this article are not readily available because although data availability may be conceivable in this context, it is not included in the ethical committee vote. Therefore patients data cannot be made publicly available even if anonymized, as they might be identified by other means. Requests to access the datasets should be directed to fasolt.meinert@med.uni-heidelberg.de.

## Author's note

Contents of this manuscript were presented as a POD at the AATS Aortic Symposium 2024 in New York City.

## References

[1] Ma WG Zheng J Dong SB Lu W Sun K Qi RD Sun’s procedure of total arch replacement using a tetrafurcated graft with stented elephant trunk implantation: analysis of early outcome in 398 patients with acute type a aortic dissection. Ann Cardiothorac Surg. (2013) 2(5):621–8. 10.3978/j.issn.2225-319X.2013.09.06 24109570 PMC3791189

[2] Poon SS Tian DH Yan T Harrington D Nawaytou O Kuduvalli M Frozen elephant trunk does not increase incidence of paraplegia in patients with acute type a aortic dissection. J Thorac Cardiovasc Surg. (2020) 159(4):1189–96.e1. 10.1016/j.jtcvs.2019.03.097 31126657

[3] Beckmann E Martens A Kaufeld T Natanov R Krueger H Rudolph L Frozen elephant trunk in acute aortic type a dissection: risk analysis of concomitant root replacement. Eur J Cardiothorac Surg. (2022) 62(4):ezac051. 10.1093/ejcts/ezac051 35134884

[4] Yoshitake A Tochii M Tokunaga C Hayashi J Takazawa A Yamashita K Early and long-term results of total arch replacement with the frozen elephant trunk technique for acute type a aortic dissection. Eur J Cardiothorac Surg. (2020) 58(4):707–13. 10.1093/ejcts/ezaa099 32236552

[5] Li Y Guo H Shi Y Liu Y Sun X. Early outcome of aortic balloon occlusion during total aortic arch replacement with the frozen elephant trunk technique for aortic dissection. Interact Cardiovasc Thorac Surg. (2020) 30(1):91–8. 10.1093/icvts/ivz229 31670767

[6] Czerny M Siepe M Beyersdorf F Feisst M Gabel M Pilz M Prediction of mortality rate in acute type a dissection: the German registry for acute type a aortic dissection score. Eur J Cardiothorac Surg. (2020) 58(4):700–6. 10.1093/ejcts/ezaa156 32492120

[7] Czerny M Grabenwoger M Berger T Aboyans V Corte D Chen A Eacts/sts guidelines for diagnosing and treating acute and chronic syndromes of the aortic organ. Eur J Cardiothorac Surg. (2024) 65(2):ezad426. 10.1093/ejcts/ezad426 38408364

[8] Nezic DG Zivkovic IS Milicic MD Milacic PA Kosevic DN Boricic MI On-Line risk prediction models for acute type a aortic dissection surgery: validation of the German registry of acute aortic dissection type a score and the European system for cardiac operative risk evaluation ii. Eur J Cardiothorac Surg. (2022) 61(5):1068–75. 10.1093/ejcts/ezab517 34915555

[9] Berezowski M Kalva S Bavaria JE Zhao Y Patrick WL Kelly JJ Validation of the geraada score to predict 30-day mortality in acute type a aortic dissection in a single high-volume aortic centre. Eur J Cardiothorac Surg. (2024) 65(2):ezad412. 10.1093/ejcts/ezad412 38109506

[10] Umana-Pizano JB Nissen AP Sandhu HK Miller CC Loghin A Safi HJ Acute type a dissection repair by high-volume vs low-volume surgeons at a high-volume aortic center. Ann Thorac Surg. (2019) 108(5):1330–6. 10.1016/j.athoracsur.2019.04.040 31158351

[11] Khan H Hussain A Chaubey S Sameh M Salter I Deshpande R Acute aortic dissection type A: impact of aortic specialists on short and long term outcomes. J Card Surg. (2021) 36(3):952–8. 10.1111/jocs.15292 33415734

[12] Mori M Shioda K Wang X Mangi AA Yun JJ Darr U Perioperative risk profiles and volume-outcome relationships in proximal thoracic aortic surgery. Ann Thorac Surg. (2018) 106(4):1095–104. 10.1016/j.athoracsur.2018.05.081 29969620

[13] Berger T Weiss G Voetsch A Arnold Z Kreibich M Rylski B Multicentre experience with two frozen elephant trunk prostheses in the treatment of acute aortic dissectiondagger. Eur J Cardiothorac Surg. (2019) 56(3):572–8. 10.1093/ejcts/ezz037 30844055

